# Is Hypertension Associated with Worse Renal Functional Outcomes after Minimally Invasive Partial Nephrectomy? Results from a Multi-Institutional Cohort

**DOI:** 10.3390/jcm11051243

**Published:** 2022-02-25

**Authors:** Rocco Simone Flammia, Umberto Anceschi, Antonio Tufano, Gabriele Tuderti, Maria Consiglia Ferriero, Aldo Brassetti, Andrea Mari, Fabrizio Di Maida, Andrea Minervini, Ithaar H. Derweesh, Umberto Capitanio, Alessandro Larcher, Francesco Montorsi, Daniel D. Eun, Jennifer Lee, Lorenzo G. Luciani, Tommaso Cai, Gianni Malossini, Alessandro Veccia, Riccardo Autorino, Cristian Fiori, Francesco Porpiglia, Michele Gallucci, Costantino Leonardo, Giuseppe Simone

**Affiliations:** 1 Urology Unit, Department of Maternal-Child and Urological Sciences, Policlinico Umberto I Hospital, Sapienza University of Rome, 00162 Rome, Italy; roccosimone92@gmail.com (R.S.F.); antonio.tufano@gmail.com (A.T.); costantino.leonardo@uniroma1.it (C.L.); 2 Department of Urologic Oncology, IRCCS “Regina Elena” National Cancer Institute, 00144 Rome, Italy; gabriele.tuderti@gmail.com (G.T.); marilia.ferriero@gmail.com (M.C.F.); aldo.brassetti@gmail.com (A.B.); michele.gallucci50@gmail.com (M.G.); puldet@gmail.com (G.S.); 3 Unit of Oncologic Minimally-Invasive Urology and Andrology, Department of Experimental and Clinical Medicine, Careggi Hospital, University of Florence, 50134 Florence, Italy; andreamari08@gmail.com (A.M.); fabridima90@gmail.com (F.D.M.); andreamine@libero.it (A.M.); 4 Department of Urology, UC San Diego School (UCSD), San Diego, CA 92103, USA; ideerwesh@gmail.com; 5 Unit of Urology, Urological Research Institute, IRCCS Ospedale San Raffaele, 20132 Milan, Italy; umbertocapitanio@gmail.com (U.C.); larcher.alessandro@hsr.it (A.L.); montorsi.francesco@hsr.it (F.M.); 6 Lewis Katz School of Medicine, Temple University, Philadelphia, PA 19140, USA; daniel.eun@tuhs.temple.edu (D.D.E.); jennifer.y.lee@temple.edu (J.L.); 7 Department of Urology, Santa Chiara Regional Hospital, Azienda Provinciale per i Servizi Sanitari (APSS), 38122 Trento, Italy; lorenzo.luciani@apss.tn.it (L.G.L.); ktommy@libero.it (T.C.); gianni.malossini@apss.tn.it (G.M.); 8 Division of Urology, Virginia Commonwealth University, Richmond, VA 23298, USA; a.veccia88@gmail.com (A.V.); ricautor@gmail.com (R.A.); 9 Department of Urology, San Luigi Gonzaga Hospital, University of Turin, 10043 Orbassano, Italy; cristian.fiori@unito.it (C.F.); porpiglia@libero.it (F.P.)

**Keywords:** hypertension, kidney neoplasm, robot-assisted partial nephrectomy

## Abstract

Background: Hypertension (HTN) is a global public health issue. There are limited data regarding the effects of HTN in patients undergoing partial nephrectomy (PN) for renal tumors. To address this void, we tested the association between HTN and renal function after minimally invasive PN (MIPN). Methods: Using a multi-institutional database (2007–2017), we identified patients aged ≥ 18 years with a diagnosis of cT1 renal tumors treated with MIPN. Kaplan–Meier plots and Cox regression models addressed newly-onset CKD stage ≥ 3b or higher (sCKD). All analyses were repeated after 1:1 propensity score matching (PSM). Results: Overall, 2144 patients were identified. Of those, 35% (*n* = 759) were yes-HTN. Yes-HTN patients were older, more frequently male and more often presented with diabetes. Yes-HTN patients harbored higher RENAL nephrometry scores and higher cT stages than no-HTN patients. Conversely, yes-HTN patients exhibited lower preoperative eGFRs. In the overall cohort, five-year sCKD-free survival was 86% vs. 94% for yes-HTN vs. no-HTN, which translated into a multivariable HR of 1.67 (95% CI: 1.06–2.63, *p* = 0.026). After 1:1 PSM, virtually the same results were observed (HR 1.86, 95% CI: 1.07–3.23, *p* = 0.027). Conclusions: Yes-HTN patients exhibited worse renal function after MIPN when compared to their no-HTN counterparts. However, these observations need to be further tested in a prospective cohort study.

## 1. Introduction

Partial nephrectomy (PN), when technically feasible, is nowadays considered the preferred surgical option for cT1 renal tumors because it ensures equivalent oncological outcomes and improved preservation of renal function when compared to radical nephrectomy (RN) [1]. However, there is a risk of ischemic damage and de novo CKD that can occur after PN [2]. For these reasons, several studies investigated modifiable surgical factors, such as decreased ischemia time and decreased parenchymal volume loss, that can minimize renal damage [3]. In addition to surgical factors, patient comorbidities such as hypertension may affect postoperative renal function [4]. Hypertension (HTN) is a global public health issue representing the number one factor in the global burden of disease [5,6]. Between the years 2011 and 2014, the prevalence of age-adjusted HTN among adults aged ≥ 20 years increased to 30.4% [7]. Moreover, hypertension is a major risk factor for chronic kidney disease, second only to diabetes [8,9]. However, the effects of HTN on renal function have mostly been shown through their association with chronic kidney disease (CKD) [10]. Conversely, there are limited data regarding the effects of HTN in patients undergoing PN [11,12,13]. To address this void, we relied on a large, contemporary, multi-institutional, minimally invasive database. We hypothesized that HTN has a negative association with postoperative renal function in patients treated with minimally invasive PN (MIPN) for cT1 renal tumors.

## 2. Materials and Methods

### 2.1. Study Population

Using a multi-institutional database (2007–2017), we identified patients aged ≥ 18 years with a diagnosis of cT1 renal mass treated with MIPN, either robot-assisted or laparoscopic. Patients with no information about hypertensive (HTN) status or patients with no available follow-up ≥1 month were excluded from the current study. HTN status was derived from patient files/medical records at each institution.

### 2.2. Statistical Analyses

First, we focused on newly-onset CKD stage ≥ 3b or higher (sCKD), as defined in previous studies [14,15]. We relied on Kaplan–Meier plots and Cox regression models addressing sCKD-free survival according to HTN status (yes vs. no). Multivariable adjustments were made. Covariates were age (one-year interval), diabetes status (yes vs. no), preoperative eGFR (continuously coded, mL/min, calculated according to CKD-epi equation [16]), RENAL nephrometry score [17] (one-point interval, 4–15) and warm ischemia time (WIT, minutes, continuously coded). Second, to address important differences in baseline patient, tumor and perioperative characteristics, we performed propensity score matching (PSM) [18]. One-to-one PSM was applied and relied on age, diabetes status, preoperative eGFR, RENAL score and WIT. After PSM, Kaplan–Meier plots and Cox regression models were re-fitted. All tests were two-sided, with a level of significance set at *p* < 0.05, and R software environment for statistical computing and graphics (version 3.4.3) was used for all analyses [19].

## 3. Results

### 3.1. Descriptive Characteristics 

Overall, 2144 patients treated with MIPN for cT1 renal tumors were identified (Table 1). Of those, 35% (*n* = 759) were yes-HTN. Yes-HTN patients were older (64 vs. 59 years, *p* < 0.001), more frequently male (67% vs. 57%, *p* < 0.001) and yielded a higher rate of diabetes (24% vs. 10%, *p* < 0.001) than their no-HTN counterparts. Yes-HTN patients were more frequently diagnosed with kidney tumors yielding higher RENAL nephrometry scores (>6, 52% vs. 45%, *p* = 0.007) and higher cT stages (cT1b, 29% vs. 23%, *p* = 0.003) than no-HTN patients. Moreover, yes-HTN patients harbored higher rates of malignant tumors relative to no-HTN patients (83% vs. 78%, *p* = 0.013). Conversely, yes-HTN patients exhibited lower preoperative eGFRs (median 80 vs. 88, *p* < 0.001). Finally, neither statistically significant nor clinically meaningful differences were recorded in other baseline assessable variables: length of hospital stay (LOS), warm ischemia tima (WIT), minimally invasive approach, postoperative complications, pT stage and surgical margin status.

### 3.2. Unmatched Analyses: Effect of Hypertension on Newly-Onset CKD ≥ 3b (sCKD)

**Figure 1:**
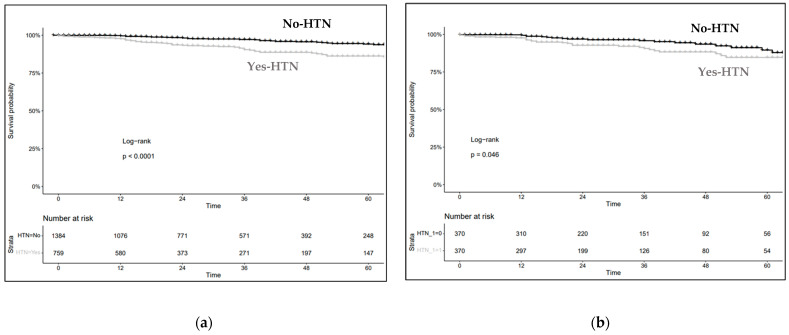
(**a**) Kaplan–Meier plots depicting sCKD-free survival according to hypertension status (yes-HTN vs. no-HTN-no) in patients treated with minimally invasive partial nephrectomy (MIPN); (**b**) after propensity score matching (PSM), Kaplan–Meier plots depicting sCKD-free survival according to hypertension status (yes-HTN vs. no-HTN) in patients treated with MIPN.

In the overall cohort, five-year sCKD-free survival was 86% vs. 94% for the yes-HTN vs. no-HTN groups (Figure 1a). These rates translated into a univariable HR of 2.48 (95% CI: 1.72–3.57, *p* < 0.001) for the yes-HTN vs. no-HTN groups (Table 2). After multivariable adjustments, an HR of 1.67 (95% CI: 1.06–2.63, *p* = 0.026) was recorded (Table 2).

### 3.3. Matched Analyses: Effect of Hypertension on Newly-Onset CKD ≥ 3b (sCKD)

In the overall cohort, PSM was applied to 759 yes-HTN and 1385 no-HTN patients. One-to-one PSM (age, diabetes status, preoperative eGFR, RENAL score and WIT) resulted in two equally sized groups of 375 females and 375 males, with no residual statistically significant differences in age, diabetes status, preoperative eGFR, RENAL score or WIT. After PSM, five-year sCKD-free survival was 85% vs. 92% for the yes-HTN vs. no-HTN groups (Figure 1a). These rates translated into an HR of 1.86 (95% CI: 1.07–3.23, *p* = 0.027) for the yes-HTN vs. no-HTN groups (Table 2).

## 4. Discussion

HTN represents a major risk factor for CKD, second only to diabetes. However, the effects of HTN on renal function have mostly been shown through their association with CKD. In fact, there are abundant studies reporting that patients with underlying CKD due to medical causes show worse renal functional outcomes and less renal recovery after PN [18]. Conversely, there are limited studies that have specifically investigated the effects of HTN on postoperative renal function in patients treated with PN [11,13]. By relying on a multi-institutional database, we aimed to clarify what role, if any, the presence of HTN should play in the clinical decision making of patients undergoing MIPN for kidney tumors.

First, we observed important differences in baseline patient and tumor characteristics. The yes-HTN patients were older, more frequently male and more often presented with concomitant diabetes than their no-HTN counterparts. Moreover, yes-HTN patients were diagnosed with bigger and more complex renal masses and harbored higher RENAL scores and higher cT stages. Finally, yes-HTN patients presented with lower eGFRs. Taken together, yes-HTN patients harbored several characteristics which predisposed them to worse postoperative renal functional outcomes.

Second, we examined the effect of HTN on postoperative renal function in the overall cohort. Here, yes-HTN patients exhibited worse postoperative renal functional outcomes than their no-HTN counterparts, even after multivariable adjustments for important confounders such as RENAL score, WIT, preoperative eGFR, age and diabetes (HR 1.67, *p* = 0.026). To account for potentially important differences in baseline patient and tumor characteristics between the yes-HTN and no-HTN patients, a more complete adjustment using either matching or weighting techniques may be required. To address this point, we relied on 1:1 PSM. After PSM, we recorded virtually the same results as after conventional analysis (HR 1.86, *p* = 0.027). Taken together, we relied on a retrospective cohort study design and observed that yes-HTN patients exhibited lower sCKD-free survival than their no-HTN counterparts. Moreover, we applied two different analytical methodologies. The first relied on multivariable adjustments for population-mix differences at baseline. The second relied on advanced matching techniques to maximally reduce population differences prior to sCKD comparisons. Both methodologies yielded virtually the same results. 

To the best of our knowledge, only two studies have previously addressed the same issue. Both Isharwal et al. and Beksac et al. reported that HTN does not impact functional recovery after PN in the short-term period. These results apparently contrast with our data. However, the current study relied on longer follow-up (60 months), a larger sample size (*n* = 2144) and a different endpoint (newly-onset CKD ≥ 3b). Hypothetically, this endpoint, together with the longer follow-up, provided us with a more generalizable picture of HTN effects in patients treated with PN because the effect of HTN is not limited to a specific time point but continues after surgery for life. Moreover, we relied on PSM in order to adjust for potentially important differences in baseline patient and tumor characteristics. 

Finally, the implications of our findings for clinical practice are several-fold. First, yes-HTN patients more likely harbored other comorbidities, such as diabetes, and presented with older ages and lower eGFRs. For this reason, patients with HTN should be better evaluated in the preoperative setting, since medical CKD is associated with worse functional outcomes [20]. In this regard, Bhindi et al. recently developed preoperative tools to predict either long-term renal function or risk of early postoperative renal failure following RN and PN, respectively [4]. HTN was included in these predictive tools, where it showed a significant contribution. Second, in patients with HTN, an effort to maximally reduce all modifiable factors affecting postoperative renal function should be made. Although this statement might appear obvious, Abouassaly et al. recently reported that PN was underutilized in patients at risk for CKD, and particularly in patients with HTN (Canadian Institute for Health Information Discharge Abstract Database, *N* = 24,579, 1998–2008) [21]. 

Despite its novelty, our study is not devoid of limitations. First, the retrospective design limited the accuracy of the data collection and the number of assessed variables. For example, we lacked data about other important comorbidities apart from HTN and diabetes as well as more specific information on HTN, such as a strict definition of HTN, time from initial diagnosis, the magnitude of the disease and the treatment received. Moreover, we did not have available data on preserved parenchyma, which is considered the main important predictor of postoperative renal function, together with WIT. Second, our study was multicenter. In consequence, different techniques for PN, as well as different perioperative management strategies, were adopted across multiple centers. However, these and all other limitations related to the multicenter, retrospective nature of this database apply equally to this study and to other similar analyses. To overcome these limitations, a prospective cohort study investigating the effects of comorbidities on postoperative renal function after PN should be launched.

## 5. Conclusions

Patients with HTN exhibited worse renal function after MIPN relative to their counterparts. However, these observations need to be further validated in a prospective cohort study that specifically addresses the role of comorbidities such as HTN in this particular setting.

## Figures and Tables

**Table 1:** Descriptive characteristics of patients undergoing minimally invasive partial nephrectomy (MIPN) stratified according to hypertensive status (yes-HTN vs. no-HTN).

Characteristic	Overall, *n* = 2144 ^1^	No-HTN, *n* = 1385 ^1^ (65%)	Yes-HTN, *n* = 759 ^1^ (25%)	*p*-Value ^2^
**Age** (years)	61 (52, 70)	59 (49, 68)	64 (56, 72)	<0.001
**Sex**				<0.001
Male	1297 (60%)	788 (57%)	509 (67%)	
**Presence of Diabetes**	320 (15%)	136 (9.8%)	184 (24%)	<0.001
**eGFR pre** (mL/min)	85 (70, 100)	88 (74, 103)	80 (63, 94)	<0.001
**LOS** (days)	4 (3, 5)	4 (3, 5)	4 (2, 5)	<0.001
**WIT** (minutes)	15 (3, 22)	15 (0, 22)	17 (10, 23)	<0.001
**Surgical Approach**				0.3
Laparoscopic	255 (12%)	157 (11%)	98 (13%)	
Robotic-assisted	1889 (88%)	1228 (89%)	661 (87%)	
**Postoperative Complications**				0.6
No	1364 (80%)	852 (79%)	512 (80%)	
Clavien–Dindo 1–2	289 (17%)	181 (17%)	108 (17%)	
Clavien–Dindo 3–5	56 (3.3%)	39 (3.6%)	17 (2.7%)	
**Clinical T Stage**				0.003
1a	1608 (75%)	1067 (77%)	541 (71%)	
1b	536 (25%)	318 (23%)	218 (29%)	
**Pathologic T Stage**				0.4
1	1688 (96%)	1062 (96%)	626 (95%)	
≥2	77 (4.4%)	45 (4.1%)	32 (4.9%)	
**Histology**				0.01
Benign	341 (20%)	230 (22%)	111 (17%)	
Malignant	1369 (80%)	823 (78%)	546 (83%)	
**Positive Surgical Margin**	100 (4.9%)	61 (4.6%)	39 (5.5%)	0.4
**RENAL Nephrometry Score**				0.01
High	169 (10%)	114 (10%)	55 (9.8%)	
Intermediate	628 (37%)	392 (35%)	236 (42%)	
Low	891 (53%)	621 (55%)	270 (48%)	

^1^ Median (IQR); *n* (%) ^2^ Wilcoxon rank-sum test; Pearson’s Chi-squared test. eGFR: estimated glomerula fitration rate; LOS: length of hospital stay; WIT: warm ischemia time.

**Table 2:** Before propensity score matching (PSM) univariable and multivariable Cox regression models addressing newly-onset CKD ≥ 3b (sCKD). After PSM Cox regression model addressing sCKD.

Cox Regression Models Addressing Newly-Onset CKD ≥ 3b (sCKD)						
	Univariable		Multivariable *		PS-Matched **	
	HR (95% CI)	*p*-Value	HR (95% CI)	*p*-Value	HR (95% CI)	*p*-Value
**HTN** (yes vs. no)	2.48 (1.72–3.57)	<0.001	1.67 (1.06–2.63)	0.026	1.86 (1.07–3.23)	0.027

* Multivariable adjustment for RENAL score, WIT, preoperative eGFR, age, diabetes status; ** 1:1 PS matching for RENAL score, WIT, preoperative eGFR, age, diabetes status.

## Data Availability

The data presented in this study are available on request from the corresponding author. The data are not publicly available due to privacy restrictions.

## Abbreviations

PN = partial nephrectomy; RN = radical nephrectomy; HTN = hypertension; MI = minimally invasive; CKD = chronic kidney disease; sCKD = newly-onset CKD ≥ 3b; WIT = warm ischemia time; LOS = length of hospital stay.
